# Neuroticism predicts the impact of serotonin challenges on fear processing in subgenual anterior cingulate cortex

**DOI:** 10.1038/s41598-018-36350-y

**Published:** 2018-12-17

**Authors:** Bettina Hornboll, Julian Macoveanu, Ayna Nejad, James Rowe, Rebecca Elliott, Gitte M. Knudsen, Hartwig R. Siebner, Olaf B. Paulson

**Affiliations:** 1 Danish Research Centre for Magnetic Resonance (DRCMR), Copenhagen University Hospital Hvidovre, Hvidovre, Denmark; 2 Center for Integrated Molecular Brain Imaging (Cimbi), Copenhagen, Denmark; 3 Child and Adolescent Mental Health Centre, Capital Region Psychiatry, Copenhagen, Denmark; 4 Neurobiology Research Unit, Copenhagen University Hospital Rigshospitalet, Copenhagen, Denmark; 5 Department of Clinical Neurosciences, Cambridge University, Cambridge, United Kingdom; 6 Division of Neuroscience and Experimental Psychology, University of Manchester, Manchester, United Kingdom; 7 Department of Neurology, Copenhagen University Hospital Bispebjerg, Copenhagen, Denmark; 8 University of Copenhagen, Faculty of Health Science and Medicine, Copenhagen, Denmark

## Abstract

The personality trait neuroticism is associated with increased vulnerability to anxiety and mood disorders, conditions linked with abnormal serotonin neurotransmission and emotional processing. The interaction between neuroticism and serotonin during emotional processing is however not understood. Here we investigate how individual neuroticism scores influence the neural response to negative emotional faces and their sensitivity to serotonergic tone. Twenty healthy participants performed an emotional face task under functional MRI on three occasions: increased serotonin tone following infusion of a selective serotonin reuptake inhibitor (SSRI), decreased serotonin tone following acute tryptophan depletion (ATD) protocol, and no serotonin challenge (control). During the task, participants performed a gender-discrimination task of neutral, fearful or angry facial expressions. Individual variations in neuroticism scores were associated with neural response of subgenual anterior cingulate cortex to fearful facial expressions. The association was however opposite under the two serotoninergic challenges. The fear-related response in this region and individual neuroticism scores correlated negatively during citalopram challenge and positively during ATD. Thus, neuroticism scores were associated with the relative impact of serotonin challenges on fear processing in subgenual anterior cingulate cortex. This finding may link to a neural mechanism for the variable therapeutic effect of SSRI treatment observed in clinical populations.

## Introduction

Impaired emotion-related processing has been associated with an increased risk for affective psychiatric illnesses, and facial expressions are linked to emotions and especially involved in social interactions^1,2^. Human and animal studies have provided cumulating evidence that serotonergic (5-hydroxytryptamine, 5-HT) neurotransmission plays a key role in processing and regulation of emotions)^3^. In healthy individuals, functional brain imaging has shown that serotonergic challenges alter the neural processing of facial expressions^4–6^, and may differentially modulate the neural response to different emotions such as anger and fear^5,7,8^. Whereas both fearful and angry faces imply threat, the type of threat is different for the two types of emotion. Where anger represents a more direct threat to the viewer and elicits avoidance behaviors, fear represents a more ambiguous threat and may at times elicit approach behaviors^9^.

In healthy individuals, acute (one dose) and extended treatment (up to seven days) with the selective serotonin reuptake inhibitor (SSRI) citalopram were shown to attenuate amygdala activation in response to negative facial expressions relative to neutral faces^5,10–12^ and to reduce attentional shifts away from aversive faces^13^. Acute SSRI was further shown to increase the neural response to happy but not to fearful faces in the amygdala^14^ whilst a facilitation in the recognition of fearful faces has also been found^15^. SSRI treatment with fluoxetine decreased the activation level significantly in the sgACC and amygdala which has been found to be significantly higher in adolescents with major depressed compared to normal controls for fearful compared to neutral facial expressions^16^.

In contrast to SSRI treatment, the ingestion of an amino acid mixture depleted of L-tryptophan, but containing large neutral amino acids yields a reversible decrease of central 5-HT synthesis. Termed Acute Tryptophan Depletion (ATD), it has shown to enhance the neural response to angry faces in a widespread neural network including frontal regions^5^, and to elicit a decreased recognition of fearful facial expressions in healthy women^17^.

Neuroticism is characterized by a tendency to worry and experience negative affect^18^. This personality trait has been associated with increased vulnerability to anxiety and mood disorders such as depression^19^. Positron emission imaging (PET) revealed an association between individual neuroticism scores and 5-HT_2A_ receptor binding in frontolimbic areas, pointing towards a link between 5-HT signaling and neuroticism^20,21^. Functional brain imaging studies have further shown that the degree of neuroticism is associated with amygdala-prefrontal connectivity in response to viewing negative facial expressions^22^. Furthermore, neuroticism has been found to correlate positively with amygdala and sgACC activation during trials of high emotional conflict, compared with low emotional conflict trials^23^ and a higher sgACC activation has been found in response to fearful faces^24^.

The aim of the present study was to explore how the individual personality trait neuroticism predicts the impact of fluctuations in central 5-HT levels on emotional processing. To address this question we investigated a cohort from the Cimbi study (Center for Integrated Molecular Brain Imaging) where neuroticism correlations were planed from the outset. We assessed how the neuronal response to negative emotional stimulation is modulated by the interplay between 5-HT level and individual neuroticism scores. We studied a group of healthy subjects with blood oxygen level dependent (BOLD) functional MRI (fMRI). The fMRI measurements of the brain were performed in a state of decreased 5-HT transmission induced by ATD, a state of increased 5-HT transmission caused by the SSRI citalopram and a normal state without either 5-HT challenge. The task required subjects to discriminate the gender of faces with fearful, angry or neutral expressions. We chose these facial expressions because, although they may both imply threat, the behavioral response is different for the two facial expressions^9^ and numerous studies indicate a different neural response to angry vs. fearful faces (see review by Fusar-Poli^7^. Our principal focus is on the contrast between the global serotonergic changes induced by ATD and SSRI challenges, in terms of the interaction between the subject variable neuroticism and emotion processing. Based on previous studies of similar serotonergic challenges, we hypothesized that there may be no main effect of drug in a heterogeneous normative cohort.

## Results

### Behavioral data

**Figure 1:**
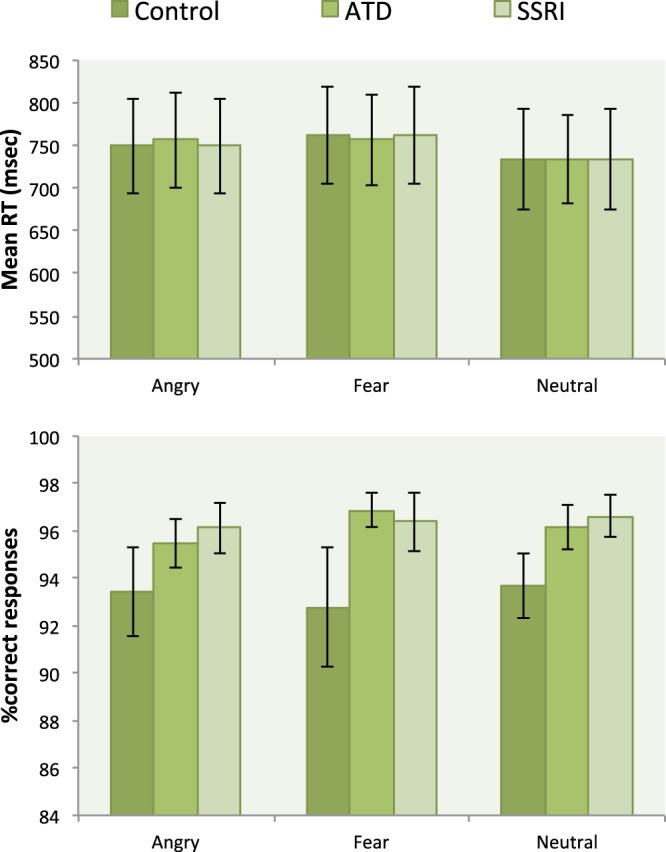
Mean reaction time (RT) and error rates as recorded during the gender-judgment task based on facial expressions in the control, ATD and SSRI challenges. RT data are presented as mean ± standard error of the mean (SEM), error rates are presented as % correct responses ± standard error of the mean (SEM).

The 3 × 3 ANOVA of the reaction time RT showed a main effect of emotion (F(1.9,40.8) = 25.5, p < 0.001), but no main effect of drug (F(1.8,37.7) = 0.91; p = 0.894) and no interaction between drug and emotion (F(2.7,56.2) = 0.568; p = 0.619). Mean RT was longer when subjects judged the gender of a fearful or angry face relative to a neutral face (F(37.7; 40.8) = 25.5; p < 0.001; Fig. 1). For the ATD challenge, mean RT was longer for angry than for neutral faces (t(21) = 2.68, p = 0.014) the same was true for fearful faces (fear: t(21) = 2.98, p = 0.007). Mean RT was longer for angry than neutral faces in the SSRI session (t(21) = 5.33, p < 0.001).

With respect to mean error rates, a 3 × 3 ANOVA with the factors 5-HT challenges (*Control, ATD and SSRI*) and emotions (*angry, fear and neutral*) showed a main effect of emotion (F(1.7, 37.7) = 34.89, p < 0.001) and drug (F(1.1, 23.9) = 6.12, p = 0.018), but no interaction between challenge and emotion. Simple t-tests revealed that error rates were overall decreased in SSRI challenge with a mean error rate of 2% compared to the control with a mean error rate of 4% for all three facial expressions (angry faces: p = 0.018, fearful faces: p = 0.026, neutral faces: p = 0.026). A comparable reduction in error rates was also found in the ATD condition with a mean error rate of 2% (fearful faces: p = 0.013, neutral faces: p = 0.053; angry faces: p = 0.051).

The ANOVA of the POMS yielded a significant effect of time for Anger/Hostility with lower scores at the end of the scanning session as compared to pre-scanning baseline (F(12) = 6.98, p = 0.022) indicating that the subjects did not remain in a high arousal state throughout the scan. Importantly, there was no significant intervention × time interaction in any of the reported mood states. Because the fMRI analyses focused on the differential effects of ATD and SSRI, we also set up a second ANOVA model with 2 × 3 factors including the two 5-HT interventions (*ATD and SSRI*) and time (*arrival, before scan and after scan*). Here we found a main effect of time, with a decrease in Vigor/Activity scores after both pharmacological interventions (F(22) = 6.61, p = 0.009), but neither a main effect of the type of intervention nor an intervention × time interaction for any of the mood states.

### Biochemical data

Baseline prolactin levels correlated highly between challenges (r = 0.80, n = 19, p < 0.001). The ATD protocol reduced the plasma ratio of tryptophan by 75% (paired t-test: before M = 49.2, SD = 10.0; after M = 12.3, SD = 12.7; t(21) = 11.2, p < 0.001) consistent with reductions in central tryptophan bioavailability^25,26^. An ANOVA of prolactin levels revealed no main effect of drug (F < 1) or time within challenge from baseline to scanning (F1, 17 = 2.86, ns).

### Neuroimaging data

Across all three conditions (ATD, SSRI, and control), the right inferior frontal gyrus as well as bilateral clusters of middle temporal gyrus stretching to the middle occipital gyrus and bilateral fusiform gyrus and amygdala showed increased activity for aversive faces relative to neutral faces (see Table 1 for peak coordinates). When separating the emotional faces, a similar pattern was found for activity that was greater for fearful than neutral faces (see Table 1 for peak coordinates). However, amygdala activity increases for angry faces compared to neutral faces were not seen (see Table 1 for peak coordinates). Neither the main effect of Challenge (SSRI and ATD) nor first level interaction between Challenge and Emotion (angry, fearful, and neutral) revealed any significant results.

**Table 1:** Peak Montral Neurogical Institute (MNI) coordinates and statistics of factorial model analysis of emotion across all challenges.

Contrast	Anatomical region	Cluster size (voxels)	x	y	z	Peak T	Peak Z	P_FWE_
Aversive faces > Neutral faces	Right middle temporal gyrus/middle occipital gyrus	402	51	−58	5	10.16	>8	<0.001
	Right fusiform gyrus	85	42	−46	−16	9.02	>8	<0.001
	Left fusiform gyrus	85	−42	−49	−16	8.70	>8	<0.001
	Left middle occipital gyrus/middle temporal gyrus	368	−30	−94	−1	8.14	7.47	<0.001
	Right amygdala	10	21	−7	−13	6.63	6.24	<0.001
	Right inferior frontal gyrus	13	54	32	5	6.02	5.72	<0.001
	Left amygdala	4	−21	−7	−16	5.66	5.41	0.0016
Angry faces > Neutral faces	Right middle temporal gyrus	184	51	−58	5	8.63	7.84	<0.001
	Right fusiform gyrus	30	42	−46	−16	7.07	6.61	<0.001
	Left fusiform gyrus	39	−42	−49	−16	7.05	6.60	<0.001
	Left middle occipital gyrus/middle temporal gyrus	158	−48	−79	5	6.86	6.44	<0.001
	Right middle occipital gyrus	38	27	−91	8	5.77	5.50	<0.001
Fearful faces > Neutral faces	Superior temporal gyrus	109	48	−40	8	7.34	6.83	<0.001
	Right fusiform gyrus	24	42	−46	−19	6.83	6.42	<0.001
	Left fusiform gyrus	36	−42	−46	−19	6.41	6.06	<0.001
	Left middle occipital gyrus	27	−30	−94	−4	6.23	5.91	<0.001
	Right amygdala	17	24	−13	−13	6.01	5.72	<0.001
	Right middle occipital gyrus	23	27	−88	2	5.81	5.54	<0.001
	Right middle temporal gyrus	7	48	−10	−16	5.63	5.38	0.0019
	Left middle temporal gyrus	9	−51	−49	11	5.58	5.34	0.0024

The table reports XYZ coordinates, T and Z values, and *p* statistical value for each cluster peak, FWE corrected for multiple comparisons at the peak-level.

### Impact of neuroticism on the responsiveness to drug challenges

**Figure 2:**
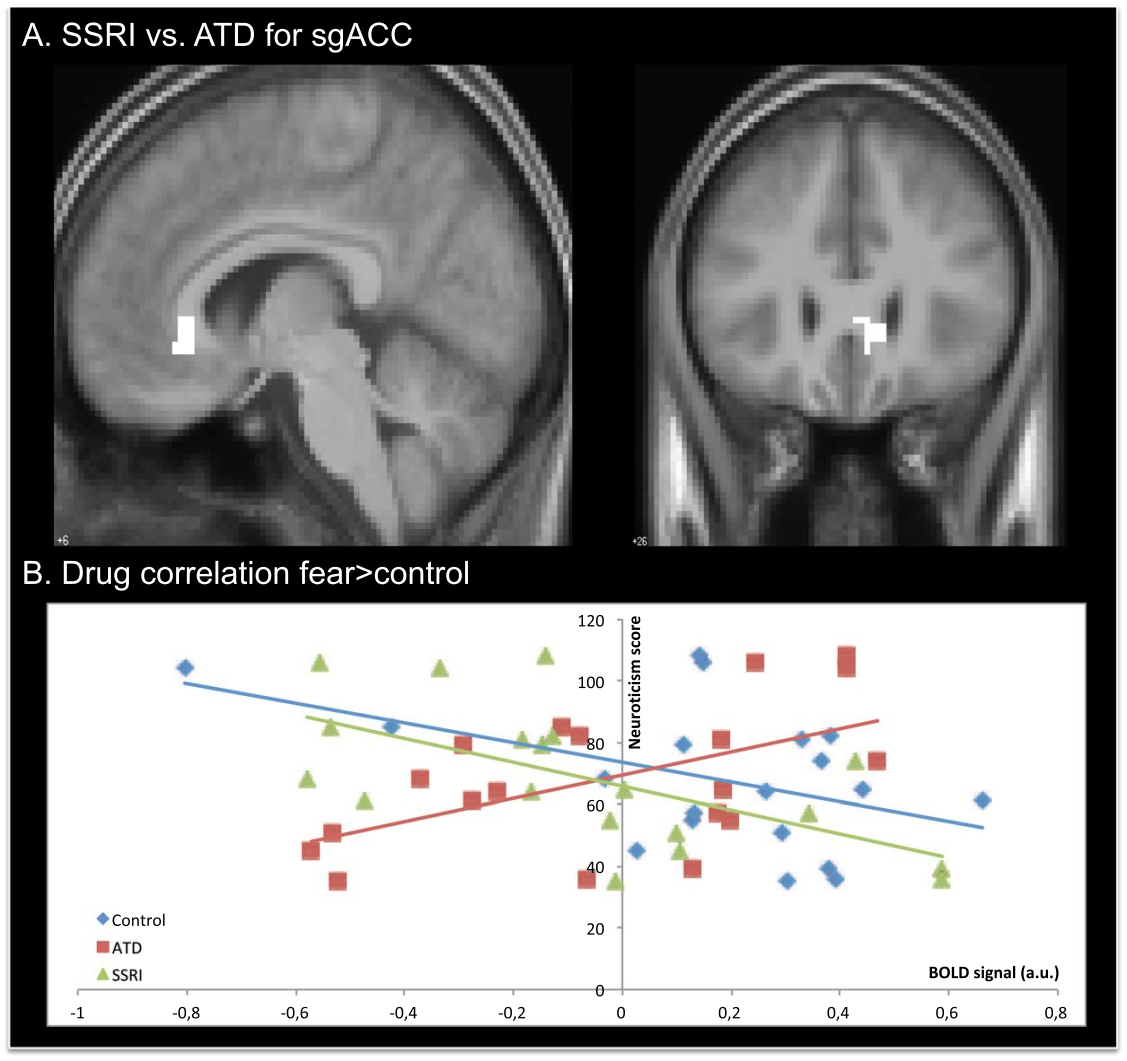
(**A**) Statistical parametric map (SPM) showing changes in the subgenual cortex (sgACC) activation for fearful face expressions during the challenges. The SPM indicate changes in BOLD signal and are thresholded at p < 0.001 (uncorrected). (**B**) Shows the correlation between the individual neuroticism scores and the BOLD response in the sgACC for SSRI, control and ATD challenges.

Individual neuroticism scores modulated the effects of drug and emotion, and their interaction. Neuroticism was negatively associated with the impact of citalopram compared to ATD (SSRI > ATD) on the neural response of the right subgenual anterior cingulate cortex (sgACC) to fearful faces (Fig. 2). The higher the neuroticism score, the lower the sgACC activity increase with SSRI compared to the ATD condition (x, y, z = 9, 26, −4, Z = 5.0, p_FWE_ = 0.015), No such relationship was seen for angry faces. A post hoc analyses excluding two subjects who’s neuroticism score were more than 5 years from day of scanning, showed an increased correlation with sgACC activation (x, y, z = 9, 26, −4, p_FWE_ = 0.009 and z = 5.1).

Post hoc analyses revealed a positive relationship between neuroticism scores and sgACC activity evoked by fearful facial expressions in the ATD challenge (Pearsons correlation; r = 0.551, p = 0.014). In contrast, we found a negative relationship between neuroticism scores and sgACC response to fearful faces in the SSRI challenge (Pearsons correlation; r = −0.611, p = 0.005).

## Discussion

Our main finding is that personality trait neuroticism alters the effect of serotonin challenges on the neural response to fearful faces. The higher the neuroticism score, the lower the sgACC activity for SSRI compared to ATD condition (Fig. 2). This is driven by a negative correlation between neuroticism and activity levels in sgACC for fearful faces for the SSRI challenge, but a positive correlation for ATD. This suggests that SSRI treatment might be more effective in subjects scoring high in neuroticism, which is in accordance with previously published material showing increased neuroticism scores to be associated with higher 5-HT_2A_ receptor binding^21^. Furthermore, neuroticism scores has been found to increase functional connectivity of the amygdala with prefrontal regions^27^. Neuroticism is a risk factor for anxiety and mood disorders^19^, possibly associated with a circuitry involving the subgenual cortex together with parts of the orbitomedial PFC, amygdala, hippocampus, striatum and thalamus^28^. Furthermore, hypothalamus, raphe, and periaqueductal gray has been shown to be anatomically connected with the subgenual cortex in the monkey brain^29^, suggesting that abnormal synaptic connections between these areas and the subgenual cortex may contribute to abnormalities in emotion processing or regulation thereof. Hall *et al*.^30^ found activation in subgenual cortex to be correlated with the severity of depressive symptoms. A recent PET study found a positive correlation between serotonergic transporter (SERT) binding and cortisol awakening response (CAR) in subgenual cortex^31^. One might speculate that the neuroticism score may have been different if the NEO-PI-R test had been done during the pharmacological challenges. The NEO-PI-R test is known to be very stable throughout the life span^32,33^ and we find it highly unlikely that the scores would have been influenced during these short-lasting pharmacological challenges. In any case the NEO-PI-R tests in the present study represents the individual baseline personality scores.

Furthermore Madsen *et al*.^27^ did a post hoc psychophysiological interaction (PPI) between the amygdala and the sgACC as reported by Pezawas *et al*.^34^ for s-allele carriers showed a higher interaction. This increased interaction between the amygdala and the sgACC may be maladaptive under severe stress, potentially underlying the increased risk for developing depression within the context of stress, for s-alleles carriers^35^.

Synthesis of serotonin (5-HT) is dependent on the precursor L-tryptophan (TRP). We included only healthy subjects and found that neuroticism scores enhance the effect of ATD as compared to SSRI. This is in accordance with previous findings, e.g. Neumeister *et al*.^36^ showing that patients with major depressive disorder showed increased BOLD fMRI activation in the orbitofrontal cortex, anterior and posterior cingulate cortices, in response to ATD. A high proportion, 50–60% of patients with major depressive disorders experience depressive like symptoms in response to ATD, whereas healthy controls seem unaffected^37^. Further, ATD can induce a transient return of depressive symptoms in some patients with remitted major depressive disorders but not in controls^36^.

The overall difference between elevated levels of free serotonin due to SSRI, and the global lowering of serotonin levels due to ATD did not reveal any main effect of drug when combining all facial expressions (neutral, angry, fear). However, in our previous study in the same group of subjects exploring the effects of 5-HT challenges on the neural response to emotional faces we have shown that an increase of serotonin levels due to acute citalopram infusion, abolished differential brain activity across aversive emotional expressions (angry and fear), whereas a reduction of serotonin levels due to ATD specifically enhanced the neural response towards angry faces, making it indistinguishable from the response to fearful faces^5^. While our current analyses did not reveal any significant main effect of either of the 5-HT challenges on neural activation compared to the control scan, we did find an increased accuracy following both ATD and SSRI interventions (Fig. 1). Furthermore, Grady *et al*.^5^, found a significant impact of ATD and SSRI on the selectivity on neural response to fearful faces, however the selectivity depended on the serotonin effect. An enhancement of the brains response to angry faces was seen with the ATD condition making the neural response similar to that of fearful faces, whereas the SSRI abolished differential brain responses across all three facial emotions (neutral, angry, fear). It is notable however, that our previous study implemented a multivariate data model in contrast with the univariate statistical analysis implemented here. Although more recent studies has used dynamic video stimuli of face processing or social attention cues (e.g.^38,39^) our stimuli were all of static emotional facial expression. Static stimuli lent it self more easily to standardization for experimental studies, including timing and controlling for confounding variable in lower level physical features, which can be more challenging in dynamic stimuli. Also, we see a significant effect of task across drugs, dismissing the notion that our facial stimuli could be a confounding factor for the lack of significant findings when contrasting the SSRI and ATD challenges with the control scan.

We found no effect of 5-HT challenges on performance of the face task in terms of RT. Therefore, the effects observed in the brain can be attributed to the interactions of serotonin challenge and the neural processing of face emotions, and not to drug effect on behavior or mood.

Although participants reported themselves to be more angry or hostile on scanning days in general, POMS scores did not correlate with any of the activation measures and accordingly, we found no evidence that the changes of mood scores had any influence on the observed brain activity.

There are methodological and pharmacological limitations to the study. Due to differences in the protocols of the two 5-HT challenges, specifically the different routes of administering the challenges and the intervals between drug administration and scanning, the drug challenges were not double-blinded. A placebo control would have been advantageous in several respects, and prevent the need to consider placebo effects or effects of IV versus no manipulations. However, drug administration and blood samples were done by a trained medical laboratory technologist to ensure uniformity, whereas data collection were done by researchers. Additionally, as mentioned in the method section, when the study was designed, the study team and ethics committee agreed that a full placebo control of the oral ATD solution and the IV infusion for SSRI challenge, would be excessive for a within-subject design. Given the heterogeneity of cerebral 5-HT_2A_ receptor binding and other genetic or personality factors relevant to inhibition, a between-subjects design might have been compromised differently, by uncertainty over the cause of differences between groups and imperfect matching. A cross-over design, albeit with a no-drug condition without blinded placebo IV/oral solutions was seen as the preferred option. Although subjects were made aware of potential side effects within the study, they were not made aware of the specific differences between the interventions or expected effects of these. Volunteers had no prior information about expected effects of the individual drugs; therefore, the task- and drug-specific observations cannot be readily accounted for by a simple placebo effect, a lack of blinding, or even an expectation or anticipation of the action of either SSRI or ATD. The only effects of the pharmacological challenges on mood, as obtained from the POMS, was on anger/hostility scores, but these were not significantly related to any of the fMRI activity measures. This would appear to rule out any general influence of the drug challenges or scanning procedures on regional task-specific brain activity. Moreover, we did not see any differences in perfusion measures between drug challenges. Although we cannot rule out some influence of these procedural differences, we suggest that the uniqueness of this dataset, allowing for the assessment of different serotonin challenges on the brain’s response to emotion in the same individuals, outweighs these limitations.

In conclusion, both lowering and increasing the serotonergic tone of the brain increased the correlation with neuroticism scores. The personality trait neuroticism were positively associated with the relative impact of serotonergic challenges on fear processing in subgenual anterior cingulate cortex, and it could be hypothesized that this could be a biomarker for neuroticism. This finding may represent a neural mechanism for the variable therapeutic effect of SSRI treatment observed in clinical populations, and supports the hypothesis that serotonin and neuroticism are related through processing of threatening emotions.

## Methods

### Participants

We recruited twenty-six right-handed healthy adults (17 males), from an already existing cohort of healthy subjects, who had all undergone psychological testing (NEO-PI-R) as well as PET imaging of the seronergic 5-HT_2A_ receptor. Twenty participants (13 males) with a mean age of 31.2 ± 6.8 years were included in the final analysis. Three participants were excluded as they did not complete all three challenges of the study and three participants were excluded due to excessive movement during MRI scan acquisition. All participants were re-interviewed prior to inclusion to the present study. None of the participants reported a history of stimulant abuse or other psychiatric or neurological disorders, nor had they ever been prescribed antipsychotic, antidepressant, or antianxiety medication. All participants had a normal neurological examination prior to inclusion.

### Ethical approval and informed consent

Written informed consent was obtained prior to study inclusion according to the declaration of Helsinki II. The study was approved by the Ethics Committee of Copenhagen and Frederiksberg, Denmark (KF 01-2006-20).

### Behavioral task

Upon arrival and immediately after scanning, participants completed a modified Danish version of the Profile of Mood States (POMS) questionnaire^40^ to assess current mood. For the ATD and SSRI sessions, participants also completed the POMS right before the MRI scan.

During fMRI, participants performed a gender judgment task on face stimuli taken from the Karolinska Directed Emotional Faces database^41^ all images used were static images of forward facing and direct gazed faces. Participants were instructed to button press with their right index or middle finger according to the gender of the face as quickly as possible. The face stimuli were unmasked color photographs shown from a frontal perspective with neutral, fearful or angry expressions. The images were presented in the middle of the screen for 1800 ms, with a 200 ms inter-trial-interval (ITI).

We employed a mixed fMRI design with alternating emotional blocks (NEUTRAL-ANGRY-NEUTRAL-FEARFUL-NEUTRAL…) showing male and female faces in equal proportion. Each block comprised of six events, which were pseudo-randomly intermixed: three to five face stimuli (average of four), and one to three (average of two) null events (fixation cross). In total, 32 blocks of neutral, and 16 blocks of each fear and angry faces, were presented over two fMRI runs separated by a short break. Each neutral face stimulus was presented twice and aversive face stimuli were presented once. Stimulus presentation and response recordings were performed using E-prime 1.2 (Psychological Software Tools, Pittsburgh, PA, USA).

### Serotonergic challenges

Participants took part in four experimental days with the challenges: SSRI, ATD, ketanserin, and a control. The focus on this particular part of the study is on the global effect of serotonin level in the brain, increased following SSRI and decreased following ATD. As ketanserin specially blocks the 5-HT_2A_ receptor the results from the receptor blocking with ketanserin has been reported elsewhere^6^. Each challenge was performed on four different days at least one week apart. The order of the challenges was randomized and counterbalanced across participants (Table 2). All MRI measurements were carried out between noon and 6 pm. Participants were informed about common potential side effects of the challenges, but not about any expected behavioral effects of the challenges. Because of considerable differences in the mode of administration across the challenges, and after advice from the Ethics Committee, a double placebo regimen was not used for the study protocol. However, apart from the pharmacological manipulation and blood sampling, the experimental procedures were the same.

**Table 2:** Counterbalanced order of scanning days for the 17 participants included in the study.

Scanning day: Subject #	1st	2nd	3rd	4th
1	Control	Ket	SSRI	ATD
2	ATD	Control	Ket	SSRI
3	SSRI	ATD	Control	Ket
4	Ket	SSRI	ATD	Control
5	Control	Ket	SSRI	ATD
6	ATD	Control	Ket	SSRI
7	SSRI	ATD	Control	Ket
8	Ket	SSRI	ATD	Control
9	Control	Ket	SSRI	ATD
10	ATD	Control	Ket	SSRI
11	SSRI	ATD	Control	Ket
12	Ket	SSRI	ATD	Control
13	Control	Ket	SSRI	ATD
14	ATD	Control	Ket	SSRI
15	SSRI	ATD	Control	Ket
16	Ket	SSRI	ATD	Control
17	Control	Ket	SSRI	ATD

The SSRI challenge was initiated prior to fMRI acquisition with a two hour intravenous infusion of citalopram at a dose of 20 mg/h to ensure a stable and sufficient transporter blocking throughout the MRI scan (others have used shorter infusion times, see e.g.^11,42^. The initial infusion was followed by a maintenance dose during fMRI acquisition of 8 mg/h (~50 mg in total).

For the ATD challenge, upon arrival on the scanning day, subjects ingested within a maximum period of 10 minutes 75 g tryptophan-free powdered mixture of essential and non-essential amino acids dissolved in water (XLYS, TRY Glutaridon, SHS International Ltd) after having kept a low protein diet the day before. fMRI acquisition was performed five hours after ingestion.

To assess a biological effect of the serotonergic challenge serum prolactin blood samples were collected three times; before challenge (citalopram or ATD administration), right before scanning start and right after the MRI scan.

For the control condition, subjects were taken directly to the scanner without any prior waiting time or (placebo) interventions.

### Neuroticism

The Revised NEO Personality Inventory (NEO-PI-R) were obtained on average 2.2 ± 2.2, max 5.8 years prior to the fMRI scans, as an individual neuroticism scores, which were the main variable of interest. The test scores are considered stable in adult life, although some long term decline in scores have been seen to occur with age^29,32,33^. However considering the relative short time (max. 5.8 years) between NEO-PI-R testing and fMRI we considered it redundant to do a time correction on the NEO-PI-R data, and as such the NEO-PI-R was not redone on our included subject on a time closer to the fMRI scans. To further support the use of the NEO-PI-R tests done earlier we reanalyzed the data excluding the two subjects with a time interval NEO-PI-R test to scan of more than five years. This resulted in an even stronger correlation to the sgACC (Z = 5.1 and pfwe = 0.009 instead of Z = 5.0 and p_FWE_ = 0.015 as reported in result section above).

The NEO-PI-R is based on the five-factor model of personality and provides metrics for broad personality dimensions of extraversion, agreeableness, conscientiousness, neuroticism, and openness to experience. It is a psychological self-reported personality inventory, which consists of 240 items. Participants indicated on a scale from 1 to 5 how well each statement fits his or her personality. The inventory was developed to test adults without overt psychopathology^32,33^. Participants completed the Danish version of the 240-item NEO-PI-R self-report personality questionnaire. The Danish translation of the NEO-PI-R has been psychometrically evaluated and normed in a standardization sample of 600 subjects^43,44^.

Each factor score is derived by adding the scores from assessment of six personality traits (facets) of each of the five personality factors and each trait score is derived by adding the scores on eight items in 0–4 Likert format.

### Magnetic resonance imaging

MR images were acquired on a 3 T Trio scanner with an eight-channel head array coil (Siemens, Erlangen, Germany). Blood oxygen level dependent (BOLD) fMRI using a T2*-weighted gradient echo spiral echo-planar (EPI) sequence with a repetition time of 2.5 s, echo time of 26 ms, flip angle of 76°, and 41 slices with a slice thickness of 3 mm and 25% gap between slices. The EPI sequence was optimized for signal recovery in orbitofrontal cortex by tilting slice orientation from a transverse toward a coronal orientation by about 30° and the use of a preparation gradient pulse^45^. A total of 156 whole-brain volumes were acquired in each of the two fMRI sessions (total 13 min). Physiological measurements of pulse (monitored with an infrared finger clip) and respiration (monitored with a chest belt) were obtained during fMRI acquisition. B0 field maps were acquired either before or after fMRI acquisition (TR = 488 ms; TE1 = 5.19 ms, TE2 = 7.65 ms; flip angle = 60°; distance factor = 25%; FOV = 240 mm; 41 slices; slice thickness = 3 mm). We additionally acquired a high-resolution 3D structural brain scan using a T1-weighted spin echo sequence (TI/TE/TR = 800/3.93/1540 ms, flip angle 9°, 1 × 1 × 1 mm isotropic resolution).

### Analysis of the fMRI data

On visual inspection of the fMRI data, artifacts (possibly due to radio frequency interferences) were identified in some slices for seven of the fMRI scan sessions (over six subjects). We therefore applied to these seven sets of functional images the slice repair utility within the ArtRepair toolbox for SPM12 (http://spnl.stanford.edu/tools/ArtRepair), which automatically detects bad slices and repairs by interpolation from slices of previous and subsequent volumes. In order to correct for B0 field inhomogeneities, the acquired B0 fieldmap was used to create a voxel displacement map (VDM) with the Fieldmap toolbox integrated within SPM12. The resulting VDM was used to unwarp the functional images during the realignment procedure for each session. Realignment was to the first functional volume and a mean functional image was created. The mean functional image was co-registered to the T1-weighted anatomical image and co-registration parameters were applied to all other functional images. The T1-weighted anatomical image was segmented using standard tissue priors and normalized to MNI (Montreal Neurological Institute) stereotactic space. The resulting deformation field image of the non-linear warping parameters for normalization from native space to MNI template space was then applied to the functional images. Lastly, normalized functional images were smoothed with a 6 mm Gaussian kernel.

The functional data from all three scanning days were analyzed in a single event-related general linear model as separate sessions. Onsets of each task event were modeled as stick functions convolved with SPM’s canonical hemodynamic response function with separate regressors for Neutral, Angry, and Fear. Each individual’s realignment parameters and their derivatives were modeled to account for head movement and subsequent spin effects^46^ Physiological noise was modeled with RETROICOR^47,48^ to obtain six respiration and four pulse regressors. The first-level contrasts for each challenge day’s Neutral, Angry, and Fear events were entered into a second-level flexible factorial design to investigate the within-group effects of challenge and emotional face processing. There were three factors modeled: “Subject”, “type of pharmacological Challenge” (3 levels: ATD, SSRI, and control), and “Emotion” (3 levels: fearful, angry and neutral). In order to verify that subjects responded to emotional stimuli, we ran within-group t-contrasts for Fear > Neutral, Anger > Neutral, and Aversive (Fear and Anger) > Neutral. We tested F-contrasts for the main effect of Challenge and the interaction effects of Emotion x Challenge. Significant findings in these were followed up, post-hoc, by their respective t-contrasts.

In the analysis of association with neuroticism scores, we ran one-sample t-tests of contrasts between challenges for aversive faces, i.e. ATD > control, SSRI > control, and SSRI > ATD for the fear events and for the anger events, separately. Neuroticism scores for each individual were entered as a covariate of interest. These analyses were conducted on 19 participants since neuroticism data was lacking for one participant.

In all tests, the significance threshold was set to a whole-brain p < 0.05, corrected for multiple comparisons with peak-level family wise error (FWE) correction, which is a conservative threshold according to recent discussions in the field^49^.

### Analysis of task performance

Behavioral data were analyzed using SPSS (version 20, Chicago, Illinois, USA). Individual scores on mood questionnaires were analyzed using a three-way repeated measures ANOVA with the within-subject factors *challenge* (ATD, SSRI, control), *mood factors* of the POMS (6 levels), and *time* of assessment relative to fMRI (arrival, before and after scan). Reaction time changes were assessed using a two-way repeated measures ANOVA with within-subject factors *challenge* (ATD, SSRI, control) and *emotion* of the face stimuli (neutral, angry, fear). The Greenhouse-Geisser method was used to correct for non-sphericity when appropriate. Conditional on significant F-values in the ANOVA, post-hoc paired t-tests were performed. Error rates were analyzed using nonparametric Wilcoxon signed-rank tests, comparing each facial expression from the control scan with the same facial expression from the relative drug challenge. Behavioral data are given as mean ± standard deviation.
